# The Relationship Between the Yo-Yo Tests, Anaerobic Performance and Aerobic Performance in Young Soccer Players

**DOI:** 10.2478/v10078-012-0081-x

**Published:** 2012-12-30

**Authors:** Barış Karakoç, Cengiz Akalan, Utku Alemdaroğlu, Erşan Arslan

**Affiliations:** 1 Ankara University, School of Physical Education and Sports, Ankara, TURKEY.; 2 Pamukkale University Schools of Sport Sciences and Technology, Denizli, TURKEY.

**Keywords:** Yo-Yo Tests, Wingate test, VO_2_max, soccer, anaerobic power

## Abstract

The purposes of this study were to determine the relationship between performance in the Yo-Yo intermittent recovery test level 1 (YIRT1), the Yo-Yo intermittent recovery test level 2 (YIRT2) and the Yo-Yo endurance test (continuous) (YET) with maximal oxygen uptake (VO_2_max) and Wingate anaerobic performance (WaNT) test results in young soccer players (age 15.00 ± 0.0 years, body height 176.3 ± 4.2 cm and body mass 68.1 ± 3.6 kg). An ergospirometry device was used during the treadmill test (TRT) to determine VO_2_max. At the end of the study, significant differences were found between the Yo-Yo tests and TRT in terms of HRmax (TRT = 195,92, YIRT1 = 197,83, YIRT2 = 198,5 YET = 198) (p > 0.05). While there were moderate correlations between VO_2_max and YIRT 1–2 performances (respectively, r = 0.56, r = 0.53), there was only a weak relationship between VO_2_max and YET performance (r = 0.43) (distance covered). There were also moderate significant negative correlations between performance in the YIRT2 and peak power measured in the WaNT (r = −0.55), although there were no significant correlations between performance in the three tests and average power. A moderate negative correlation was found between performance in the YIRT2 and Fatigue index (FI) (r = −0,66). In conclusion, the YIRT2 may be a more suitable field test for determining both aerobic and anaerobic performance in soccer players.

## Introduction

Soccer performance is not only affected by technical skills, a player’s physical capacity is known to exert a major influence on his match performance (Little and Williams, 2007). The activities involved in soccer are of intermittent nature, with changes every 3–5 s, resulting in repeated high-intensity spells of play (Krustrup et al., 2006; Little and Williams, 2007). A soccer game involves jumping, shooting, challenges, turns, dribbles, sprints, controlling the ball under pressure, running at different speeds, and sliding tackles; both aerobic and anaerobic energetic pathways are used during games (Açıkada et al., 1998; Stolen et al., 2005). The recovery capacity of players during repeated high-intensity bouts is closely related to the development of aerobic capacity (Tomlin and Wenger, 2001; Castagna et al., 2008). Moreover, players’ high intensity movements are positively associated with their anaerobic energy pathways (Castagna et al., 2006). The outcome of a match may be determined by the aerobic and anaerobic capacity of players and it is therefore important to evaluate their aerobic and anaerobic capacity (Bangsbo et al., 2008).

The assessment of physical capacities of athletes is one of the most important issues in modern sports. Coaches and sport scientists use field and laboratory tests for screening candidates, in selection procedures, or to monitor the efficacy of training regimes (Norkowski, 2002). Numerous field tests have been developed to assess the physical capacities of athletes. Yo-Yo tests have rapidly become one of the most extensively studied shuttle run tests in sports science, due to their specificity and practicality (Krustup et al., 2003). These tests have also been applied to assess players’ abilities to repeatedly perform high-intensity exercise (Bangsbo et al., 2008) in many team sports such as soccer (Krustup et al., 2003; Aziz et al., 2005), basketball (Castanga et al., 2006) and rugby (Atkins, 2005) and it is thought that Yo-Yo tests are one of most effective field-based means of assessing soccer player’s endurance performance (Aziz et al., 2005).

Many studies have examined the relationship between Yo-Yo tests performance and VO_2_max using an oxygen analyzer, but the results of these studies present a scattered picture (Bangsbo et al., 2008). While some studies have found only weak correlation between VO_2_max and Yo-Yo test performance (Aziz et al., 2005; Castagna et al., 2006), other studies have shown a moderate to strong correlation (Krustrup et al., 2003; Thomas et al., 2006).

Few studies have examined the relationship between Yo-Yo test performance and anaerobic fitness. Castagna et al. (2006) found no correlation between counter movement jump and Yo-Yo Endurance Test (YET) or Yo-Yo Intermittent Recovery Test Level 1 (YIRT1) performance. Similarly, Krustrup et al. (2006) found no relationship between Yo-Yo Intermittent Recovery Test Level 2 (YIRTL2) and repeated sprint performance. The most commonly used test of anaerobic fitness is the Wingate anaerobic test (WaNT), which is a laboratory based cycle ergometer test (Hoffman et al., 2000). However, no previous study has compared Yo-Yo tests performance with WaNT performance, nor have any studies examined the relationships between the results of different Yo-Yo tests. It is thought that one of the Yo-Yo tests may meet the requirements of simultaneous stimulation of the aerobic and anaerobic energy system (Krustrup et al., 2006; Bangsbo, 1994). Therefore, the objectives of the current study were threefold a) to determine the relationship between performances in YIRT1, YIRT2 and YET and VO_2_max, b) to determine the relationship between Yo-Yo test and WaNT test results, c) to examine the differences in heart rate responses to Yo-Yo tests and TRT in young soccer players.

## Material and Methods

### Subjects

Twelve soccer players voluntarily participated in the study. The mean measurements gathered were as follows: age 15.00 ± 0.0 years, body height 176.3 ± 4.2 cm and body mass 68.1 ± 3.6 kg. The subjects were informed about the possible risks and benefits of the study and gave their informed consent to participate in this study, which was approved by the Clinical Research Ethical Committee of Ankara University. The study was conducted over a 2-week period, during which the players did not participate in any other training or matches. All players were recruited from the same team and had been playing competitively for at least two years. They were familiarized with the tests’ protocols and had undergone the YIRT1, YIRT2, YET, TRT and WaNT at least once prior to the study.

### Procedures

The five test trials were conducted as separate sessions with 2-day intervals between tests. On day 1, body composition measurements were taken and the participants completed a test session on a treadmill (Cosmed, Gambettola, Italy) to determine maximal oxygen uptake (VO_2_max); on day 2, the participants completed a battery of tests that examined anaerobic physical performance (WaNT); on days 3, 4, 5 the YIRT1, YIRT2 and YET were performed randomly. The YIRT1, YIRT2, YET trials were conducted on the same facilities (synthetic pitch) and all tests were performed between 10:30 and 12:30. Before the players undertook the tests they were instructed to exert maximal effort and were verbally encouraged to run for as long as possible. The standardized warm-up for the YIRT1, YIRT2 and YET trials consisted of 3 minutes of running the 20m distance back and forth at a set pace (i.e. 8.0 km/h) with the help of “beep” sounds; for the TRT trials, it consisted of 3 minutes of running on a treadmill at 8 km/h. This was followed by 5 minutes of stretching, focusing on the lower limb muscles (Aziz et al., 2005). During the TRT, expired gases were analyzed using a breath-by-breath automated gas-analysis system (Fitmate Pro; Cosmed, Italy). The flow, volume, and gas analyzer were calibrated before each player’s test according to the manufacturer’s instructions. Heart rate data were stored using HR monitors (Polar Electro OY, Kempele, Finland) throughout the tests. The stored data were transferred to computer and filtered by Polar Precision Performance Software^™^ (PPP4, Finland). The highest HR measurement was recorded as HRmax. The temperature and relative humidity at the test site were consistent throughout the study, ranging between 25.4–27.6 ºC and 51.3–53.7%, respectively. Each player completed all of the tests within the two-week period.

### Maximal Oxygen Uptake (TRT)

The treadmill exercise testing was performed to voluntary exhaustion on a motorized treadmill (Cosmed, Gambettola, Italy). All tests were performed under standardized conditions in a stable laboratory environment. Each player warmed-up on the treadmill (Venus, HPCosmos, Germany) for 3 min at 8.0 km·h^−1^ and followed this with 5 min of stretching the lower limbs (Aziz et al., 2005). For each subject, the test commenced with three minutes of running at 8.0 km·h^−1^ at zero gradient, followed by speed increases of 2 km·h^−1^ for the next 2 minutes. Thereafter, gradient was systematically increased by 2% every minute until a maximum of 12% was attained. If termination was not achieved by this time, then increases of 1 km·h^−1^ for each following 1-minute stage until exhaustion. This procedure was used by Aziz et al. (2005) in a similar study.

### The Wingate Anaerobic Test (WAnT)

The Wingate Anaerobic Test (WAnT) was conducted using a mechanically braked cycle ergometer (834 E, Monark, Vansbro, Sweden). The WAnT test was administered for 30 seconds. The subjects warmed up for 5 minutes at a pedaling rate of 50 rpm against no load, after which they rested for 5 min. They were then instructed to pedal as fast as they could. When the pedaling rate reached approximately 160–170 rpm, the resistance was applied and subjects continued pedaling as fast as possible for 30 s. Subjects were verbally encouraged during the test. Peak power and mean power was calculated automatically by the Wingate Anaerobic Test computer program (Kin-işler et al., 2008; Inbar et al., 1996). A fatigue index (FI) was calculated by using the following equation (Inbar et al., 1996).

Equation 1 FI=[(Peak Power Output-Min Power Output)/Peak Power Output×100]

### The Yo-Yo intermittent recovery tests (YIRT1-YIRT2)

Both YIRT1 and YIRT2 consist of repeated 20-m runs back and forth between the starting, turning, and finishing lines at a progressively increased speed, which is controlled by audio beeps from a tape recorder. When the subjects failed twice to reach the finishing line in time, the distance covered was recorded as the test result (Krustrup et al., 2003; Bangsbo, 1994). YIRT 1 has 4 running bouts at 10–13 km·h^−1^ and another 7 runs at 13.5–14 km·h^−1^, whereafter it continues with stepwise 0.5 km·h^−1^ speed increments after every 8 running bouts (i.e., after 760, 1080, 1400, 1720 m, etc.) until exhaustion (Krustrup et al., 2003). YIRT 2 test started at a speed of 13 km·h^−1^, which increased by 2 km·h^−1^ after the first stage and by 1 km·h^−1^ after the second stage, afterwards it continued with stepwise 0.5 km·h^−1^ speed increments after every stage until exhaustion (Krustrup et al., 2003).

### The Yo-Yo endurance test level (YET)

The Yo-Yo Endurance Test level (continuous) is a variation of the Yo-Yo test series. The YET consists of repeated 2 × 20 m runs back and forth between the starting, turning, and finishing line at a progressively increased speed controlled by audio beeps from a tape recorder (Bangsbo, 1994). When the participant stops, the final speed and the number of performed 20 m distances at this speed are recorded, including the last run.

### Statistical Analyses

The data are reported as means and standard deviations. Before using parametric tests, the assumption of normality was verified using the Shapiro-Wilk test. A one-way repeated-measures analysis of variance was performed on heart rate responses in the YET, YIRT 1–2 and TRT. A Bonferroni Post Hoc test was applied to make a pairwise comparison between YET, YIRT 1–2 and TRT tests. The Pearson product moment correlation coefficient (r) was used to determine the relationships between Yo-Yo test performance, measured VO_2_max in the TRT and anaerobic physical performance. The level of statistical significance was set at p < 0.05.

## Results

Table 2 shows the results of YIRT1, YIRT2, YET, TRT and WaNT tests.

Table 3 shows the correlations between performances (in terms of distance covered) in the three tests and the measured VO_2_max obtained from TRT and WaNT test performances for the 12 players. There were weak correlations between performance in the YET and VO_2_max, whereas moderate correlations were found between performance in the YIRT1, the YIRT2 and VO_2_max obtained in the TRT. Moreover, there were moderate significant correlations between performance in the YIRT2 and peak power obtained in the WaNT. In contrast, there were no significant correlations between performance in any of the three tests and average power obtained in the WaNT. Finally, there were moderate negative correlations between performance in the YIRT2 and FI, whereas no correlations were found between performance in the YIRT1 or the YET and FI.

Table 4 shows the correlations between performances (in terms of distance covered) in the Yo-Yo tests for the 12 players. No correlations were found between performance in the YET, in the YIRT1 and in the YIRT2.

**Figure 1:**
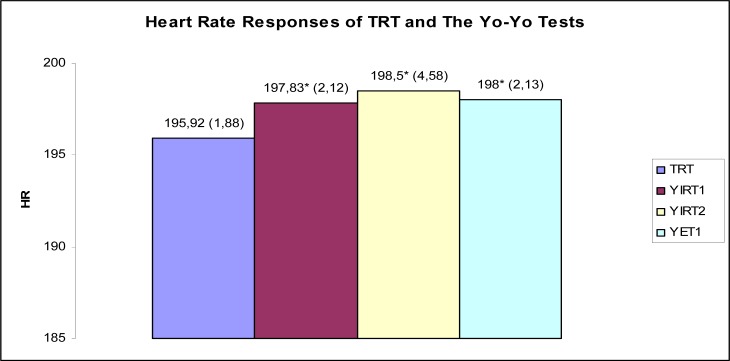
Heart rate responses of TRT and the Yo-Yo tests *significantly different from the TRT, p < 0.05; HRmax = Maximal heart rate. YET= Yo-Yo Endurance Test level 1, YIRT1= Yo-Yo Intermittent Recovery Test level 1, YIRT2= Yo-Yo Intermittent Recovery Test level 2, TRT= Treadmill test

Figure 1 shows the heart rate responses measured during the YET, YIRT1–2 and TRT for the 12 players. TRT HRmax values are significantly different from YIRT1–2 and YET HRmax values (p<0.05)

## Discussion

The measurement of VO_2_max using gas and ventilation analysers requires expensive exercise ergometers, laboratory conditions, and trained personnel, not to mention medical staff in attendance (Leibetseder et al., 2002; Stickland et al., 2003), so it may not be appropriate for team sports where testing every player will take time away from training (Stickland et al., 2003; Aziz et al., 2005). For these reasons, there is increased interest in predictive tests (Aziz et al., 2005) and sports scientists have focused on comparing these tests with each other. The major finding of this study is that, while moderate relationships were found between measured VO_2_max and distance covered in the YIRT1 and YIRT2, only a weak correlation was found between distance covered in the YET and measured VO_2_max. Krustrup et al. (2003), Thomas et al. (2006) and Rampini et al. (2010) found that YIRT 1 distance correlated strongly with VO_2_max (r = .71, r = .83, and r = 0 .78, respectively). In contrast, only weak correlations were found between distance covered in the YIRT1 and VO_2_max by Rampini et al. (2010). However, Castagna et al. (2006) found no correlation between YIRT1 performance and VO_2_max. Thus, the relationship between the YIRT1 performance and VO_2_max is unclear (Bangsbo et al., 2008).

This study found moderate relationships between measured VO_2_max and YIRT2 distance covered. Few studies have previously examined the relationship between YIRT2 performance and VO_2_max. While Rampini et al. (2010) found a weak correlation between YIRT2 performance with VO_2_max, Krustrup et al. (2006) reported a moderate correlation between YIRT2 performance and measured VO_2_max values (r = .56). Considering the results of these studies and our own, it seems that the prediction of VO_2_max from YIRT1–2 results may not be accurate (Bangsbo et al., 2008).

Another finding of this study is that of a weak correlation between YET performance and measured VO_2_max values for the subjects. While no previous study has compared YET performance and VO_2_max, the YET is similar to the MST in that they both have a continuous test design, and the results of previous studies examining the relationship between VO_2_max and MST performance suggest that the MST provides a valid prediction of VO_2_max (Leger and Gadaoury, 1989; Ramsbottom et al., 1988; Aziz et al., 2005). A plausible explanation for the apparently anomalous finding of only a weak correlation between YET and VO_2_max could be that these tests operate at different speeds.

One of the important findings of this study is its comparison of HRmax in the YIRT 1–2, the YET and the TRT. Heart rate (HR) monitoring is the most popular indirect method of estimating intensity of exercise and it also seems to be the most practical and low-cost method (Murayama and Ohtsuka, 1999). Previous studies have not generally found differences in terms of HRmax measured during the Yo-Yo tests and in different treadmill tests (Stickland et al., 2003; Aziz et al., 2005; Krustrup et al., 2006; Castagna et al., 2006). Metaxas et al. (2005) also found no significant differences in HRmax values between the Yo-Yo continuous and intermittent tests as well as 2 maximal exercise treadmill tests with continuous and intermittent protocols. In contrast, we found significant differences between HRmax values measured in the TRT and those measured in each of the Yo-Yo tests. This finding is of interest considering that, while the differences between the Yo-Yo intermittent endurance test HRmax and TRT HRmax found by Aziz et al. (2005) were not significant, the TRT HRmax values were still 5 beats per minute lower than the Yo-Yo test HRmax values. These results suggest that the Yo-Yo tests might be more suitable for determining HRmax than the TRT.

The Wingate anaerobic test (WaNT) is the gold standard for evaluating anaerobic capacity. We found moderate relationships between peak power and FI measured in WaNT and YIRT2 performance, which examines the ability to perform repeated high-intensity exercise with a high rate of anaerobic energy turnover (Krustrup et al., 2006). On the other hand, correlations were not found between average power as measured by the WaNT and any of the Yo-Yo tests results in this study. Similarly, Krustrup et al. (2006) did not find correlations between the YIRT2 test results and sprint performance or repeated sprint performance. They also found no correlations between the YIRT2 test results, muscle enzymes, and fiber-type distribution and concluded that no single factor determined a subject’s ability to perform this type of exercise. However, Castagna et al. (2006) found a significant correlation between YIRTl and vertical jump performance and they reported that performance during high-intensity intermittent exercise such as YIRT tests is influenced by maximal muscular power. As can be seen from these conflicting findings, insufficient number of studies have been conducted on relationships between Yo-Yo test and anaerobic performance. Moreover, no previous study has examined correlations between WaNT and Yo-Yo test performance. Thus it seems that further research is needed.

## Conclusion

Our results suggest that Yo-Yo tests could be used interchangeably to determine HRmax. YIRT2 may be more suitable to characterize soccer players’ intermittent endurance performance (Aziz et al., 2005) and this test may provide a more effective field-based assessment of both aerobic and anaerobic performance in soccer players.

## Figures and Tables

**Table 1:** Speeds of the Yo-Yo tests

**YIRT1 (km/h)**	**YIRT2 (km/h)**	**YET (km/h)**
10.0	13.0	8.0
11.5	15.0	9.0
13.0	16.0	10.0
13.5	16.5	10.5
14.0	17.0	10.8
14.5	17.5	11.0
15.0	18.0	11.3
15.5	18.5	11.5
16.0	19.0	11.8
16.5	19.5	12.0
17.0	20.0	12.3
17.5	20.5	12.5
18.0	21.0	12.8
18.5	21.5	13.0
19.0	22.0	13.3

YET= Yo-Yo Endurance Test level, YIRT1= Yo-Yo Intermittent Recovery Test level 1, YIRT2= Yo-Yo Intermittent Recovery Test level 2

**Table 2:** The results of tests

VO_2_max (ml/kg/min)	YIRT 1 Distance (m)	YIRT 2 Distance (m)	YET Distance (m)	WaNT peak power (watt)	Want average power (watt)
59.95± 1.23	2730.75± 159.38	1208.33± 89.22	2086.67± 128.30	719.12± 79.20	550.93± 37.06

**Table 3:** Correlations between measured vo_2_max, WaNT test performance and Yo-Yo tests distances

	VO_2_max (ml·kg^−1^·min^−1^)	Average Power (watt)	Peak Power	FI
YIRT1 Distance (m)	0.56*	0.27	−0.04	−,39
YIRT2 Distance (m)	0.53*	−0.13	−0.55*	−,66*
YET Distance (m)	0.43*	0.17	0.05	−,12

VO_2_max = Maximal oxygen uptake,; FI= Fatigue index YET= Yo-Yo Endurance Test level, YIRT1= Yo-Yo Intermittent Recovery Test level 1, YIRT2= Yo-Yo Intermittent Recovery Test level 2

* p < 0.05

**Table 4:** Correlation between performances in respective Yo-Yo tests

	YIRT2 Distance (m)	YET Distance (m)
YIRT1 Distance (m)	,52	,48
YIRT2 Distance (m)		,27

YET= Yo-Yo Endurance Test level, YIRT1= Yo-Yo Intermittent Recovery Test level 1, YIRT2= Yo-Yo Intermittent Recovery Test level 2

* p < 0.05
